# Recent Progress on Poly(3,4‐Ethylenedioxythiophene):Poly(Styrenesulfonate) Bioelectrodes

**DOI:** 10.1002/smsc.202300008

**Published:** 2023-04-24

**Authors:** Xiaojia Du, Leyi Yang, Nan Liu

**Affiliations:** ^1^ Beijing Key Laboratory of Energy Conversion and Storage Materials College of Chemistry Beijing Normal University Beijing 100875 China; ^2^ Beijing Graphene Institute (BGI) Beijing 100095 China

**Keywords:** bioelectrical signals, bioelectrodes, PEDOT:PSS, skin electronics

## Abstract

Sensing bioelectrical signals is of great significance to understand human disease. Reliable bioelectronic interface is the guarantee of high‐quality bioelectrical signals. The unique electrochemical property and the mixed ionic and electrical conductivity of poly(3,4‐ethylenedioxythiophene):poly(styrenesulfonate) (PEDOT:PSS) make it an ideal material for the skin/tissue–electronic interface. However, pristine PEDOT:PSS‐based devices cannot meet the requirements for practical use. Toward this end, herein, the development of PEDOT:PSS‐based electrodes and their most recent advances in sensing bioelectrical signals are summarized. First, the generation mechanism of bioelectrical signals is introduced in detail. Then, according to the characteristics of bioelectrical signals, the requirements of bioelectrodes are discussed. Next, representative achievements for improving conductivity, stretchability, and stability of PEDOT:PSS are introduced. Bioelectrical signals such as electromyogram (EMG), electrocardiogram (ECG), electrooculogram (EOG), and electroencephalogram (EEG) are successfully recorded by these PEDOT:PSS‐based electrodes. Finally, a brief summary is provided, and the opportunities and challenges are also discussed.

## Graphical abstract

The development of poly(3,4‐ethylenedioxythiophene):poly(styrenesulfonate) (PEDOT:PSS)‐based electrodes and their most recent advances in sensing bioelectrical signals are summarized The generation mechanism of bioelectrical signals, the requirements of bioelectrodes, representative achievements of improving conductivity, stretchability and stability of PEDOT:PSS, and recent application of epidermal and implantable PEDOT:PSS electrodes are reviewed, and the opportunities and challenges are discussed.

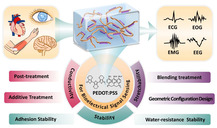

## 1 Introduction

Sensing bioelectrical signals paves a way for knowing the health and mental status, providing important clinical information about subjects’ physiological and psychological activities.^[^ ^1^ ^]^ Common bioelectrical signals include electromyogram (EMG), electrocardiogram (ECG), electrooculogram (EOG), and electroencephalogram (EEG). EMG is conducive to explore the activities of nerves and muscles. ECG signals, originating from heart, show the information related to cardiac function. EOG can help to recognize eye diseases. EEG is capable of showing complex neural activities in the brain.^[^ ^2^, ^3^, ^4^, ^5^ ^]^ Furthermore, bioelectrical signals could integrate with human–machine interfaces (HMI), such as prosthetic control, emotion recognition, eye movement tracking, virtual reality (VR), and augmented reality (AR).^[^ ^6^, ^7^, ^8^, ^9^ ^]^

So far, various bioelectrodes have been developed for recording bioelectrical signals, but it is still challenging to acquire high‐quality, stable, and long‐term signals.^[^ ^10^, ^11^ ^]^ Human skin/tissue will deform during body motion, which means that human skin/tissue has to withstand strain. Conventional bioelectrodes are mainly gel electrodes (Ag/AgCl electrodes) and dry electrodes made of metals (e.g., Au, Ag, Cu). Due to the mechanical mismatch with human skin/tissue, they are unable to continuously recording signals. In addition to, long‐term use of such electrodes would cause skin irritation. Stretchability is a critical property to ensure bioelectrodes to work normally despite the deformation of human skin/tissue. Lots of interest is devoted to the fabrication of stretchable electrodes.^[^ ^12^, ^13^, ^14^, ^15^, ^16^ ^]^ These electrodes need to possess matched modulus to adapt to the uneven surface of human skin/tissue. In addition to the mechanical properties, excellent conductivity, adhesion to skin, and biocompatibility are necessary. Furthermore, breathability and self‐healing are also the properties people pursuing for.^[^ ^17^ ^]^

**Figure 1:**
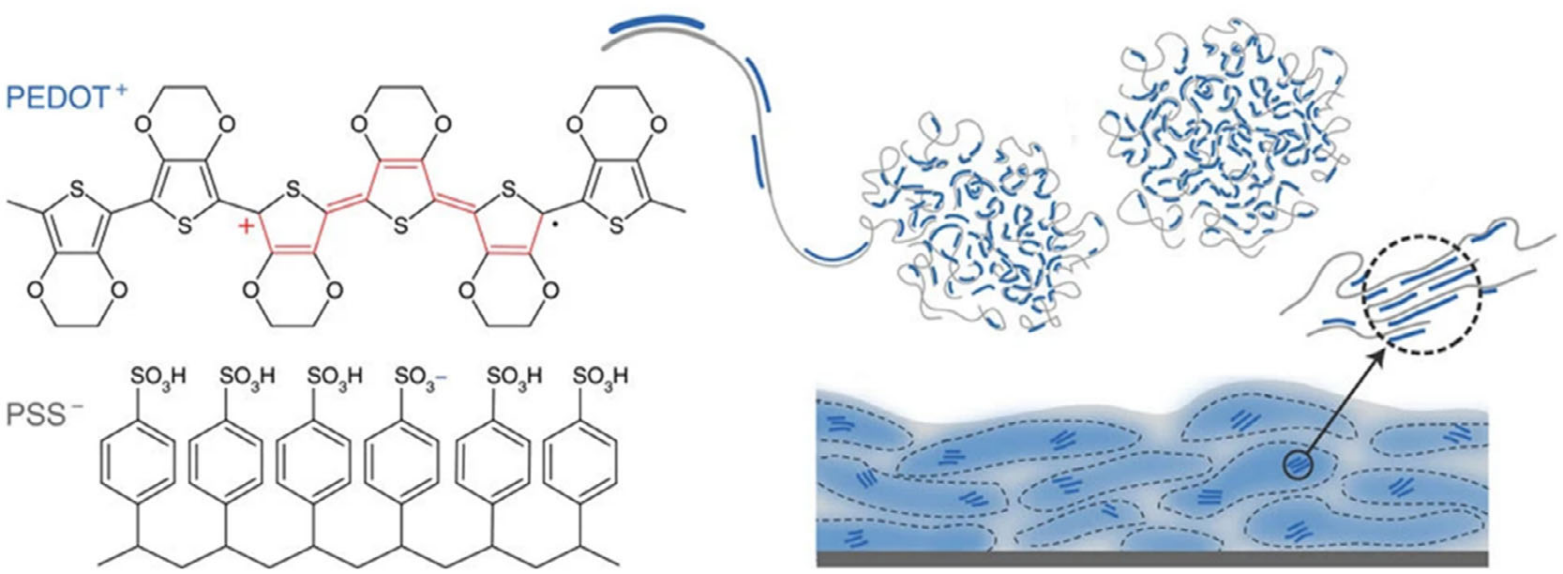
Structure and morphology of PEDOT:PSS. Reproduced with permission.^[^ ^29^ ^]^ Copyright 2016, Springer Nature.

Emerging materials used for bioelectrodes include conductive polymers, conductive hydrogels, carbon materials (e.g., graphene, carbon nanotubes), liquid metals, elastomer composites, etc.^[^ ^18^, ^19^, ^20^, ^21^ ^]^ Conductive polymers are kinds of organic conjugated polymers with heterocycle, whose conductivity stems from conjugated main chains of delocalized electron/hole. By combining the advantages of both metals and polymers, conductive polymers are easy for fabrication and structure modification. Among them, poly(3,4‐ethylenedioxythiophene) (PEDOT) has attracted heightened interest due to its excellent conductivity, optical transmittance in the visible region, thermostability, relatively low redox potential, etc. Nevertheless, its insolubility in water hinders further development. It can be overcome by introducing polyelectrolyte like poly(styrenesulfonate) (PSS) into PEDOT matrix. PSS acts as both dopant and stabilizer by a charge balance mechanism. Since the problem of insolubility in water has been solved, PEDOT becomes the most successful commercial polyelectrolyte.^[^ ^22^ ^]^ **Figure** **1** shows the structure of PEDOT:PSS.

PEDOT:PSS has become the competitive one among the next‐generation bioelectrode materials due to its adjustable conductivity, high optical transmittance, biocompatibility, and ease of processing. However, PEDOT:PSS has some functional defects, which restrict its practical application. Researchers proposed many strategies to enhance the properties of PEDOT:PSS and developed a sequence of PEDOT:PSS derivatives.^[^ ^23^ ^]^ For now, the reviews related to PEDOT:PSS mostly focus on photovoltaic, energy sources, and electrochemistry.^[^ ^24^, ^25^, ^26^ ^]^ Few reviews discuss from the perspective of bioelectrical signal sensing. Thus, this review aims to summarize recent advances in the applications of PEDOT:PSS for sensing bioelectrical signals. First, the generation mechanisms of bioelectrical signals, collection methods, and characteristics are introduced. Moreover, the tactics proposed to improve the performance of PEDOT:PSS are reviewed. Furthermore, according to their working environments, the application of epidermal and implantable PEDOT:PSS bioelectrodes is introduced. Finally, the current challenges and future opportunities for the development of PEDOT:PSS bioelectrodes are discussed.

## 2 Bioelectrical Signal

Bioelectricity is a fundamental characteristic of vital activities. Various electric currents and electric potentials would be generated during the vital activities of life. By collecting and analyzing bioelectrical signals, the functional condition of human tissue can be known, which is helpful for the assessment of physical health and clinical treatment.

**Figure 2:**
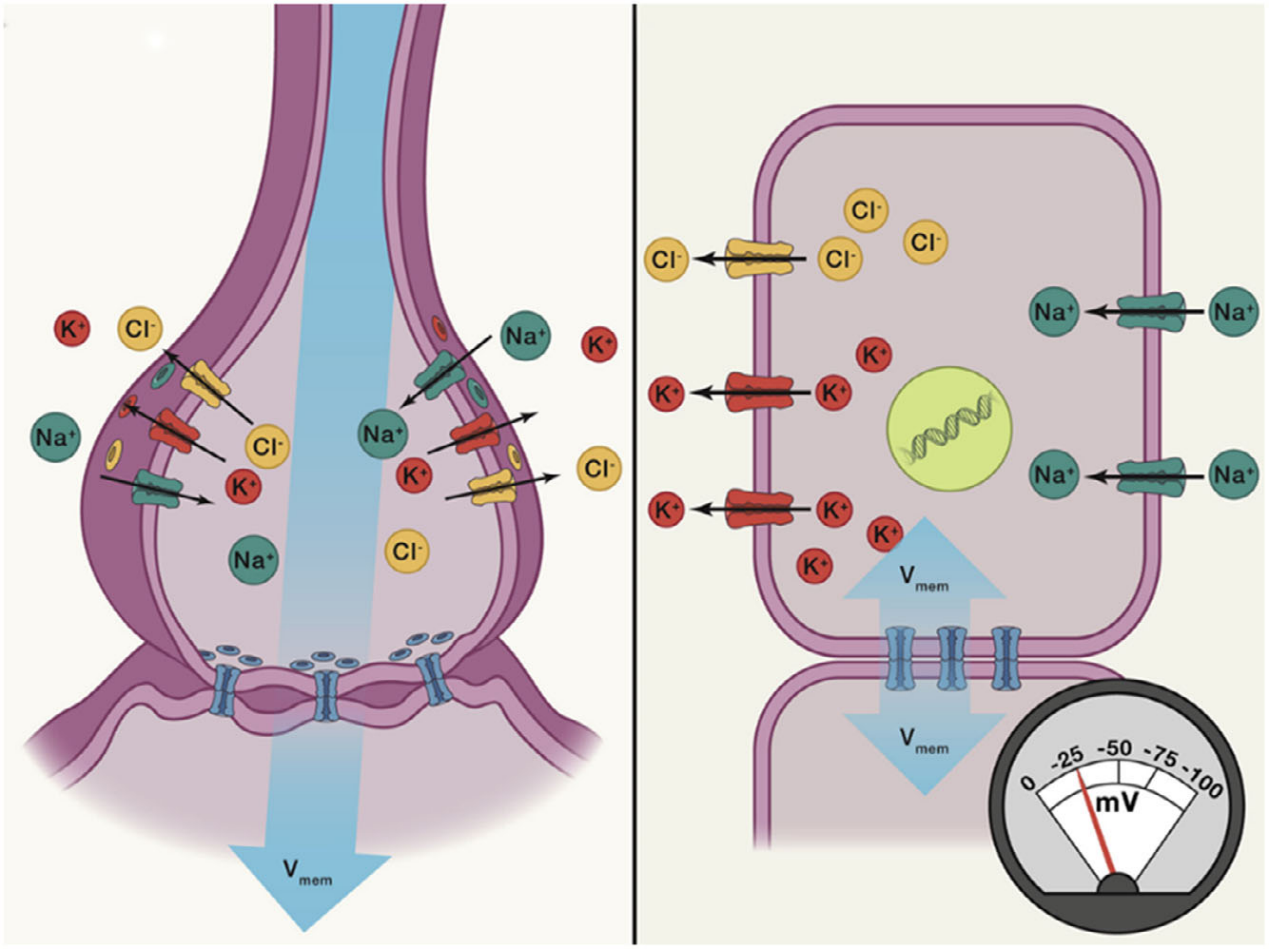
Generation of bioelectrical signals. Left) Neural bioelectric dynamics. Right) The ancient mechanisms present in cells. Reproduced with permission.^[^ ^27^ ^]^ Copyright 2021, Elsevier.

Electronic activities in a biological system are essentially the ionic activities of electrolytes. Since electrons cannot be the charge carrier in the electrolytic tissue medium, the electrocommunication between excitable cells mainly depends on ion flow (**Figure** **2**).^[^ ^27^ ^]^ The most fundamental element of bioelectrical activities is composed of ion flow or action potentials (APs) peak in excitable cells. In the resting state, the imbalance of concentration between intracellular K^+^ and extracellular Na^+^ generates potential differences (positive inside and negative outside). The transmembrane potential maintains a negative value (≈−60 to −75 mV) in the resting state, which is also called the polarization state. When cells are stimulated, a few Na^+^ ion channels open allowing Na^+^ to enter, which causes concentration difference and generates local potential. When the potential fluctuates beyond the threshold voltage (mostly −50 to −55 mV), plenty of Na^+^ ion channels open allowing a rapid influx of Na^+^ into the cells, which causes the negative membrane potential to decrease and turn to positive potential. When up to about +30 to +40 mV, the transmembrane potential repolarizes to the resting potential by slow efflux of K^+^. These processes generate APs on membranes and in surrounding extracellular fluids.^[^ ^28^ ^]^ In addition to the ionic currents, the transmission of electrophysiological signals is also controlled by chemical signals, which are mediated by the release of neurotransmitters such as dopamine and acetylcholine. Neurons communicate via the mechanism between the membranes of synapses, which connect the axon of a neuron with the dendrite of another one. There are many vesicles filled with neurotransmitters near the presynaptic area, which would be released into the synaptic cleft to send APs toward the postsynaptic area. Then, chemical substances diffuse to the acceptor sites on postsynaptic membrane to change the permeability of Na^+^, which would change the postsynaptic potentials. The formation of postsynaptic potentials and the generation of APs both involve complex interactions stimulated by ions and chemical substances, which are the main bioelectrical activities for regulating neurons.^[^ ^25^, ^26^, ^27^, ^28^, ^29^, ^30^ ^]^

### 2.1 Common Bioelectrical Signals in Human

Common bioelectrical signals in human include EMG, ECG, EOG, EEG, etc. EMG is for studying skeletal muscle, whose generation is related to the excitatory conduction of nerves. The impulses of motor neurons to muscles cause the contraction of skeletal muscles. EMG records the electricity generated during muscle contraction, which could be utilized clinically to diagnose neural and neuromuscular problems. Some research laboratories also use EMG to study biomechanics, motion control, gait analysis, neuromuscular physiology, dyskinesia, gesture control, and physical therapy.^[^ ^31^, ^32^ ^]^

ECG reflects the electrical activities of myocardial cells. The stimulation of myocardial cells induces ions to flow inside and outside the membranes and triggers depolarization and repolarization of myocardial cells. A sequence of harmonious electrical stimulated impulses inside the heart excite the muscular cells of the atria and ventricles to let the heart contract and relax rhythmically and maintain the human circulatory system working normally. The typical waveforms of ECG consist of P waves, QRS complex, T waves, and U waves. Health state of heart and cardiac diseases, such as arrhythmia and myocardial ischemia, could be accessed by analyzing ECG in clinical application.^[^ ^33^, ^34^ ^]^

EOG derives from the potential difference between cornea and retina, which could be regarded as the dipole composed of positively charged cornea and negatively charged retina. During eye movements, like moving from the center to the surrounding, the direction of dipole would change, leading to a change of potential field. Therefore, it could also induce the changes in the amplitude of electrical signals. Eye movements could be tracked by analyzing these changes. EOG could be used to examine retinal function and reflect sleep patterns, the state of brain, drowsiness, and eye fatigue.^[^ ^35^, ^36^ ^]^

EEG reflects the activities of a large number of nerve cells. EEG signals are very weak. Common EEG signals include *δ* wave (0.5–3 Hz), *θ* wave (4–7 Hz), *α* wave (8–13 Hz), and *β* wave (14–30 Hz). The electrical activities of the cerebral cortex could be classified as spontaneous EEG activity and evoked EEG activity. Spontaneous EEG is dominated by human emotion, while evoked EEG needs external stimulation. Noted that EEG is usually collected from the scalp. If the signals come from cortical surface, it would be called electrocorticogram (ECoG). EEG is usually clinically applied to diagnose epilepsy. Considerable researchers use it to recognize emotion as well.^[^ ^37^, ^38^ ^]^

### 2.2 Bioelectrical Signal Sensing

**Figure 3:**
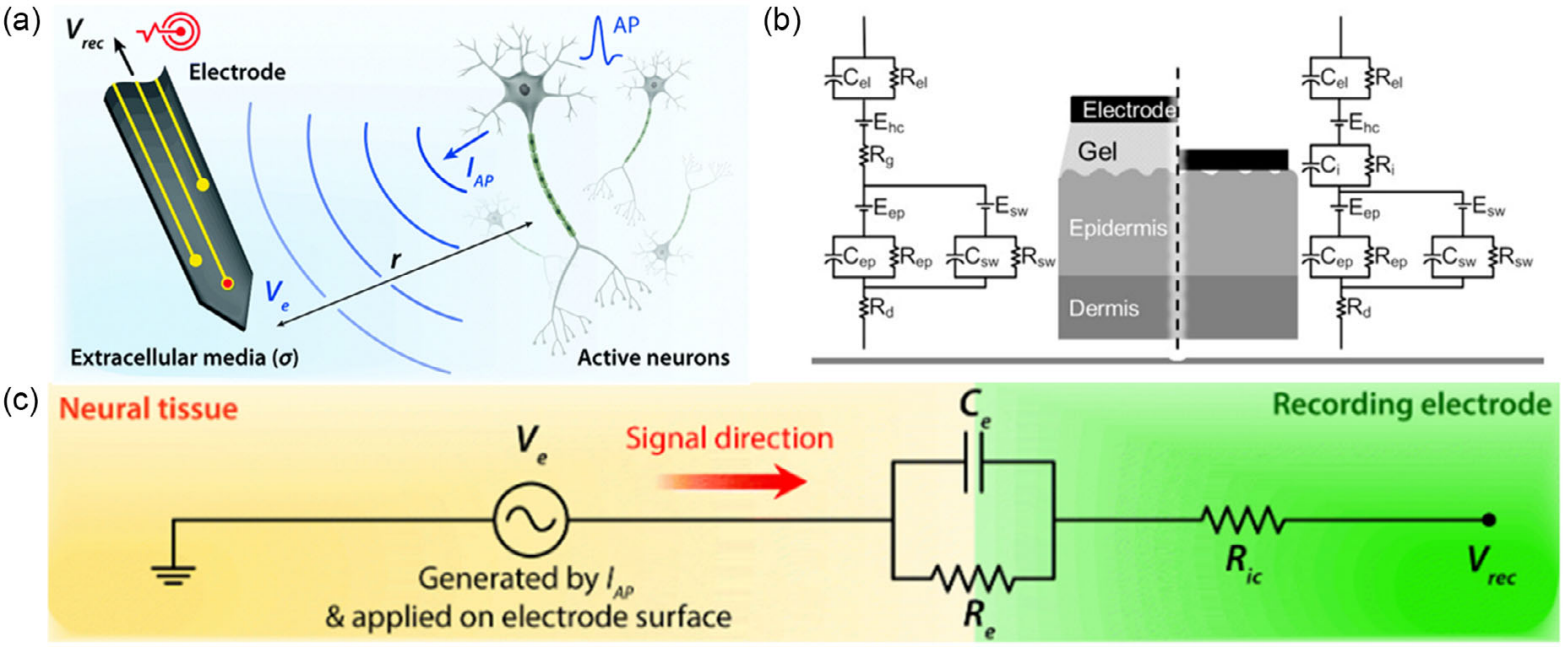
Bioelectronic activity on tissue–electrode interface. a) Schematic illustration of bioelectrical signal recording. b) Equivalent circuit model of electrode–skin interface for gel electrodes and dry electrodes. c) Equivalent circuit model of tissue–electrode interface for bioelectrical signal recording. a,c) Reproduced with permission.^[^ ^28^ ^]^ Copyright 2019, Royal Society of Chemistry. b) Reproduced with permission.^[^ ^43^ ^]^ Copyright 2013, Springer Nature.

Bioelectronic interfaces need to be established to detect bioelectrical signals. It is a general term for a variety of biological integrated electrodes that communicate with biological systems. The bioelectronic interfaces could not only be established on human skin but also in vivo. The bioelectronic activities in biological tissue–electrode interfaces involve ionic and electronic interactions at various length scales. As shown in **Figure** **3**a, *V*_e_ means the potentials generated by electrical activities of neurons (extracellular potential and local field potential generated by the APs of single neuron). Once extracellular potential or local field potential reaches recording electrodes, it would be transmitted through the electrolyte–electrode interfaces. Figure 3c shows the equivalent circuit model. At tissue–electrode interfaces, electrolyte–electrode interfaces are regarded as the parallel circuit of the electrical double layer (EDL) capacitance *C*_e_ and the leakage resistance *R*_e_. *V*_REC_ is the output of bioelectrical signals with the interconnect resistance *R*_IC_.^[^ ^28^ ^]^ Once extracellular potential or local field potential reaches recording electrodes, it would be transformed through the electrolyte–electrode interfaces.

#### 2.2.1 Electrode Requirements

The design and fabrication of high‐performance bioelectronic interfaces pursue low interface impedance and conformal adhesion. The former is essential for the capture of high‐fidelity bioelectrical signals, and the latter ensures tight contact between electrode and the uneven biological tissue surface.

The signal‐to‐noise ratio (SNR) is usually used to evaluate the quality of bioelectrical signals. The higher the SNR, the higher the signal quality. However, there exists a great deal of noisy interferences on skin–electrode interfaces. Electrodes with high conductivity are beneficial for improving SNR due to high charge carrier density and surface electric displacement field.^[^ ^39^ ^]^ Thus, it is significant to enhance the conductivity of electrodes.

Stretchability, another critical factor of bioelectrodes, can also be understood as the ability to withstand a large degree of deformation. Human skin could endure about 15% deformation without irreversible damage and could even survive a larger degree of deformation in different places. For instance, the failure strain of human back skin ranges from 37% to 71% during the quasistatic test. The failure strain of forehead and arm ranges from 27% to 59% under dynamic load.^[^ ^17^ ^]^ When electrodes endure bending, twisting, and stretching, the mechanical strain exerted on devices is quite possible beyond the fracture strain of electrode materials (≈1% for metal and ≈5% for conductive polymer).^[^ ^40^, ^41^ ^]^ Therefore, bioelectrodes are expected to possess certain stretchability to accommodate different measuring locations.

Adhesion to human skin/tissue is of great importance for bioelectrodes. Young's modulus of human skin ranges from 20 to 100 kPa, and neural tissue is softer (<10 kPa).^[^ ^42^ ^]^ Conventional rigid metal electrodes could not form conformal contact with skin due to mechanical mismatch. This unconformability with skin would generate high impedance during detection, which would lower signal quality. They would even detach during motion, which would cause the complete loss of signals. At present, the developed bioelectrodes could be classified as gel electrodes and dry electrodes. For gel electrodes, the gel on electrode–skin interfaces ensures the tight contact between electrodes and skin. However, ease of drying hinders gel electrodes from continuously collecting electrophysiological signals. As for dry electrodes, the biggest challenge is stable adhesion to skin. Figure 3b shows the electrode–skin interface equivalent circuit models of gel and dry electrodes, respectively.^[^ ^43^ ^]^ If electrodes could not conformally contact with skin (on the right of Figure 3b), the parallel resistance composed of equivalent resistance *R*_*i*_ and equivalent capacitance *C*_*i*_ would increase, resulting in a large contact impedance. The equation of conformal contact energy *U*_conformal_ could be written as

(1) $$U_{\text{conformal}}=U_{\text{skin}}+U_{\text{bending}}+U_{\text{adhesion}}$$

Among them, *U*_skin_ is the elastic energy of skin, which is independent of electrode materials. *U*_bending_ is the bending energy of electrode materials, and *U*_adhesion_ is the adhesion energy between electrodes and skin.^[^ ^44^ ^]^ Based on this equation, the skin–electrode interfaces could be optimized by decreasing Young's modulus of materials, reducing thickness, and enhancing adhesion.

The biocompatibility of electrodes is necessary. Past researches have indicated that commercial Ag/AgCl electrodes would cause skin stimulation and irritation due to the gel used to ensure adhesion. The electrodes contact directly with human skin/tissue may cause health problems as well.^[^ ^45^ ^]^ Therefore, it is significant to develop stretchable electrodes with biocompatibility for monitoring bioelectrical signals for a long time without irritation.

Electrodes with high spatiotemporal resolution broaden the detection area and improved measurement accuracy. For now, most sensors still record bioelectrical signals through a single channel, which usually consists of three electrodes: measuring, reference, and ground electrodes. Single‐channel signal detection can only provide information about the area covered by electrode. Human muscle/organ activities are complex, so there are certain limitations in the practical application of single‐channel sensors. High‐density array electrodes can cover a wider area of human skin/tissue. The high spatiotemporal resolution electrical signals provided by multichannel detection are helpful for obtaining abundant and comprehensive information on neuromuscular function. This can be used for neuromuscular function rehabilitation.^[^ ^46^ ^]^ In disease diagnosis, it can be used to accurately locate heart lesion areas. In addition, the development of HMI, brain–computer interfaces, etc., also needs a large number of electrodes.^[^ ^47^ ^]^ Therefore, from single channel to multichannel detection is a development direction of bioelectronic interfaces.

In addition to the properties mentioned above, breathability, transparency, portability, and self‐healing are also the targets people chasing for. Human skin would sweat more evidently during sports. In this case, signals are apt to drift. Therefore, breathability is an essential consideration for bioelectrodes.^[^ ^48^ ^]^ Transparency, on one hand, has esthetic effects and on the other hand, can be used to conceal or monitor the real‐time status of human skin/tissue in specific scenes. When monitoring neural activities in brain (such as EEG and ECoG), transparent electrodes can reduce the optical artifacts of EEG signals.^[^ ^49^ ^]^ Portability provides comfort when wearing the bioelectrodes. The self‐healing electrodes could mimic the self‐healing function of human skin/tissue.

## 3 Poly(3,4‐Ethylenedioxythiophene):Poly(Styrenesulfonate) Electrodes

PEDOT:PSS is one of the most representative conductive polymers, which has been commercially produced. It has good solubility in water, excellent biocompatibility, high transparency, and flexibility. Due to the solution processibility and compatibility with various film‐forming technologies, it is easy to achieve low‐cost and mass production of thin film electronic devices. However, the conductivity of pristine PEDOT:PSS‐based electrodes is very low (<1 S cm^−1^), and the inherent elongation at break of the as‐cast PEDOT:PSS films is about 2%.^[^ ^26^ ^]^ The high hydrophilicity of PEDOT:PSS film makes it unstable in humid environments. Dedoping of PEDOT:PSS would happen when exposed to alkaline environment, which damages the electrical properties.^[^ ^50^ ^]^ To overcome the above shortcomings, researchers combined various technologies and proposed different strategies, including introducing additives, posttreatments, and combining them with various materials. The strategies for enhancing PEDOT:PSS conductivity, stretchability, and stability will be discussed in detail as follows.

### 3.1 Improving Conductivity of Poly(3,4‐Ethylenedioxythiophene):Poly(Styrenesulfonate) Electrodes

#### 3.1.1 Additive Treatments

**Figure 4:**
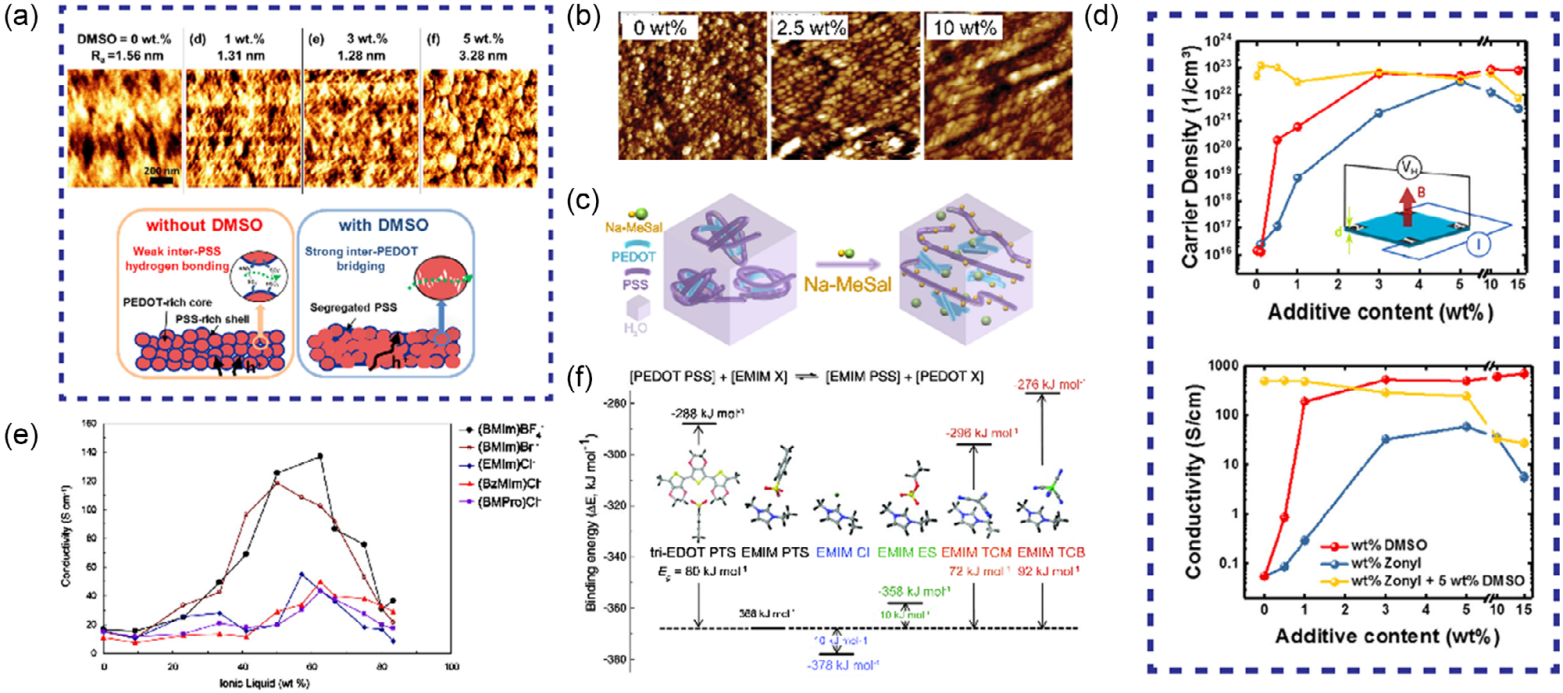
Improving the electrical conductivity of PEDOT:PSS‐based electrodes. a) AFM phase images of PEDOT:PSS layer with varying DMSO amounts of 0, 1, 3, and 5 wt%, respectively (top), and the debonding mechanism (bottom). Reproduced with permission.^[^ ^53^ ^]^ Copyright 2016, American Chemical Society. b) STM images of PEDOT:PSS with different sorbitol concentrations. Reproduced with permission.^[^ ^56^ ^]^ Copyright 2008, Wiley‐VCH. c) Schematic diagram of the conductivity enhancement mechanism of PEDOT:PSS with Na–MeSal. Reproduced with permission.^[^ ^60^ ^]^ Copyright 2021, American Chemical Society. d) Effect of the DMSO, Zonyl FS‐300, and their mixture on the electrical properties of PEDOT:PSS: Conductivity (top), carrier concentration (bottom). Reproduced with permission.^[^ ^63^ ^]^ Copyright 2019, American Chemical Society. e) Conductivity versus the amount of various ILs wt% in PEDOT:PSS films. Reproduced with permission.^[^ ^68^ ^]^ Copyright 2007, American Chemical Society. f) Binding energy diagrams of different combinations. Reproduced with permission.^[^ ^69^ ^]^ Copyright 2016, Wiley‐VCH.

The addition of polar organic solvent, surfactant, and ionic liquid (IL) to PEDOT:PSS could effectively improve the conductivity of PEDOT:PSS. The polar organic solvents with high boiling points such as dimethyl sulfoxide (DMSO), *N*‐methylpyrrolidone (NMP), *N,N*‐dimethylformamide (DMF), and ethylene glycol (EG) are widely used.^[^ ^51^ ^]^ Lim et al. improved its conductivity by three orders of magnitude by adding DMSO to PEDOT:PSS. The screen effect induced by polar solvents changed the conformation of PEDOT chains from coiled to more extended structure, which promoted interchain coupling and charge transport between PEDOT.^[^ ^52^ ^]^ Lee et al. found that doping with DMSO not only improved the conductivity of PEDOT:PSS, but also enhanced the film cohesion.^[^ ^53^ ^]^ As shown in **Figure** **4**a, adding 3 wt% DMSO can simultaneously improve conductivity and cohesion. Wei et al. found that the addition of EG improved crystallinity and order of PEDOT in films, thus increasing carrier mobility.^[^ ^54^ ^]^ Pasha et al. increased the conductivity of PEDOT:PSS to 199 S cm^−1^ by doping EG and successfully applying to gas sensors.^[^ ^55^ ^]^ Sorbitol can also be used as a dopant to improve the conductivity of PEDOT:PSS. Scanning tunneling microscopy (STM) shows that the addition of sorbitol makes PEDOT form elongated clusters, allowing charge carrier transitions to occur between clusters (Figure 4b).^[^ ^56^ ^]^

Surfactants can be classified as ionic and nonionic. Ionic surfactants include sodium dodecyl sulfate (SDS), sodium *p*‐toluenesulfonate (TsONa), sodium dodecylbenzene sulfonate, etc.^[^ ^57^ ^]^ The mechanism is that anionic surfactants can replace PSS^−^ as antianion of PEDOT chain, which makes the twisted structure of PEDOT chain disappear. This conformational transition enhances the conductivity of polymers.^[^ ^58^, ^59^ ^]^ Similarly, Liu et al. doped PEDOT:PSS with sodium 3‐methylsalicylate. The PSS chain dissociated from PEDOT:PSS due to the formation of very weak acid and PSS‐Na (Figure 4c), which enhanced the conductivity of PEDOT:PSS to 584.2 S cm^−1^.^[^ ^60^ ^]^ Nonionic surfactants include poly(ethylene glycol) (PEG), poly(vinyl alcohol) (PVA), *p*‐tert‐octylphenol (Triton X‐100), fluorine‐containing surfactants Zonyl FS‐300, etc.^[^ ^57^ ^]^ Wang et al. studied the influence of PEG with different concentrations and molecular weights on the conductivity of PEDOT:PSS in detail. They concluded that PEG formed hydrogen bonds with sulfonic acid group of polystyrene sulfonic acid (PSSH), thereby weakening the electrostatic interaction between PEDOT and PSS.^[^ ^61^ ^]^ Sun et al. first doped PEDOT:PSS with PEG and then posttreated with water to enhance the conductivity to 1415.7 ± 21.2 S cm^−1^.^[^ ^62^ ^]^ The phase separation of PEDOT:PSS induced by Zonyl FS‐300 can effectively improve the conductivity of PEDOT:PSS. Dauzon et al. achieved higher carrier density with the help of synergistic effect of Zonyl FS‐300 and DMSO (Figure 4d).^[^ ^63^ ^]^ Triton X‐100 is also a surfactant commonly used to promote conductivity.^[^ ^64^, ^65^ ^]^ In addition to enhancing conductivity, surfactants can also improve the wettability of PEDOT:PSS on hydrophobic substrates.^[^ ^66^, ^67^ ^]^

ILs are salts entirely composed of cations and anions, the physicochemical properties of which are determined by electrostatic interactions between cations and anions. They feature great chemical and thermal stability, nonvolatility, and wide electrochemical windows. As shown in Figure 4e, five ILs with different proportions are added to PEDOT:PSS solution, respectively, including 1‐butyl‐3‐methylimidazolium tetrafluoroborate (bmim)BF_4_, 1‐butyl‐3‐methylimidazolium bromide (bmim)Br, 1‐ethyl‐3‐methylimidazolium chloride (emim)Cl, 1‐benzyl‐3‐methylimidazolium chloride (bemim)Cl, and 1‐butyl‐1‐methylpyrrolidium chloride (bmpyr)Cl, which improved the conductivity of PEDOT:PSS with phase separation between PEDOT and PSS.^[^ ^68^ ^]^ Kee et al. proved that the degree of counterion exchange between ions and PSS^−^ could be controlled by adjusting the electrostatic interaction between ions, which reduced the π–π stacking distance of PEDOT and made it highly ordered (Figure 4f).^[^ ^69^ ^]^ Under optimal conditions, the conductivity of PEDOT:PSS can be increased by 5000 times to about 2100 S cm^−1^ at most. The fibrous structure and more interconnected nanostructures occurred after IL doping confirmed the phase separation between PEDOT and PSS. Via density functional theory (DFT) calculation, Izarra et al. concluded that the most effective ion pair was the one with the lowest binding energy, and proposed a new ion pair based on this.^[^ ^70^ ^]^

Other additives include salt solutions, acids, and nanocarbon materials. Xia et al. studied the doping effects of CuCl_2_, AgNO_3_, InCl_3_, LiCl, NaCl, MgCl_2_, and NiCl_2_ on PEDOT:PSS and found that salt solutions with “soft” metal ions could increase the conductivity of PEDOT:PSS by two orders of magnitude.^[^ ^71^ ^]^ Inorganic acids such as H_2_SO_4_, HCl, and HClO_4_ can improve the electrical conductivity of PEDOT:PSS as well.^[^ ^57^ ^]^ After a systematic study, Zhang et al. found that acids with high boiling points, low acid dissociation constants, and low anion softness can effectively induce phase separation between PEDOT and PSS, which promoted effective charge transport.^[^ ^72^ ^]^

#### 3.1.2 Posttreatments

Posttreatment methods are immersing PEDOT:PSS films into specific solvents/solvent baths. At present, posttreatment methods can be classified as organic solvent posttreatment and acid posttreatment. Common organic solvents include DMSO and EG.^[^ ^73^, ^74^ ^]^ Some studies found that DMSO posttreatment can improve the conductivity of PEDOT:PSS more effectively than adding DMSO.^[^ ^75^ ^]^ Ouyang group investigated the EG posttreatment mechanism. The enhancement of conductivity was due to the conformational transition of PEDOT chains from benzene to quinone.^[^ ^76^ ^]^ Ouyang group also systematically studied the effects of hexafluoroacetone, cyclohexanone, formaldehyde, acetaldehyde, and other solvents.^[^ ^77^, ^78^ ^]^ Posttreatment with methanol solution can also increase the conductivity of PEDOT:PSS films by four orders of magnitude. This is due to the interaction between methanol with high dielectric constant and PSS, which generates a shielding effect and promotes the phase separation between PEDOT and PSS.^[^ ^79^ ^]^

Acid posttreatment can significantly improve the electrical conductivity of PEDOT:PSS films. Kim et al. used H_2_SO_4_ to posttreat PEDOT:PSS film. A highly crystalline structure was observed through atomic force microscopy (AFM). During the treatment process, the structure of PEDOT:PSS was rearranged. The subsequent washing process not only removed excess H_2_SO_4_ but also took away a large amount of PSS. This enhances the conductivity to 4380 S cm^−1^, which is the highest conductivity in this system.^[^ ^80^ ^]^ Milder acids, such as formic acid and mesylate, have also been used to improve the conductivity of PEDOT:PSS.^[^ ^81^, ^82^ ^]^

In addition, surface modification of PEDOT:PSS can also improve the conductivity in a more environmental‐friendly way, which avoids the use of solvents. Common methods include surface modifiers coating, laser microannealing, hot pressing, and mild plasma.^[^ ^83^, ^84^, ^85^, ^86^, ^87^, ^88^, ^89^ ^]^ Due to its spatial selectivity, laser microannealing technology can achieve patterning and high conductivity of PEDOT:PSS simultaneously. By controlling the irradiation intensity below the damage threshold, phase separation would be induced between PEDOT and PSS. The nanostructures of PEDOT cores would rearrange and crystallize.^[^ ^86^, ^87^, ^88^, ^89^ ^]^ Yun et al. enhanced the conductivity of PEDOT:PSS by three orders of magnitude through laser illumination. The mechanism lies in the difference in photon absorption between PEDOT and PSS. PEDOT cores were heated up and their heat energies were transferred to the PSS nanoshells, which are then fragmented. The physical contacts between adjacent PEDOT cores were enhanced and the transport ways were improved, leading to a conductivity as high as 932 S cm^−1^.^[^ ^86^ ^]^ Won et al. developed an ultrafast digital patterning process for PEDOT:PSS by using a continuous‐wave 532 nm laser. With the addition of gold nanoparticles (AuNPs), the interaction between PEDOT:PSS and laser was intensified, and the laser‐induced phase separation PEDOT:PSS hydrogel can achieve a conductivity of 560 S cm^−1^.^[^ ^88^ ^]^

### 3.2 Improving Stretchability of Poly(3,4‐Ethylenedioxythiophene):Poly(Styrenesulfonate) Electrodes

To form a tight contact between electrodes and human skin/tissue, the mechanical properties of functional materials should be similar to those of human skin/tissue. Despite its flexibility, PEDOT:PSS has very limited extensibility due to its rigid conjugated main chains and strong interaction between chains. Therefore, it cannot be used directly as a stretchable electrode. The strategies for preparing PEDOT:PSS stretchable electrodes can be classified into two main categories, namely, geometric structure design and blending with plasticizers, IL, polymers, etc.

#### 3.2.1 Geometric Configuration Design

**Figure 5:**
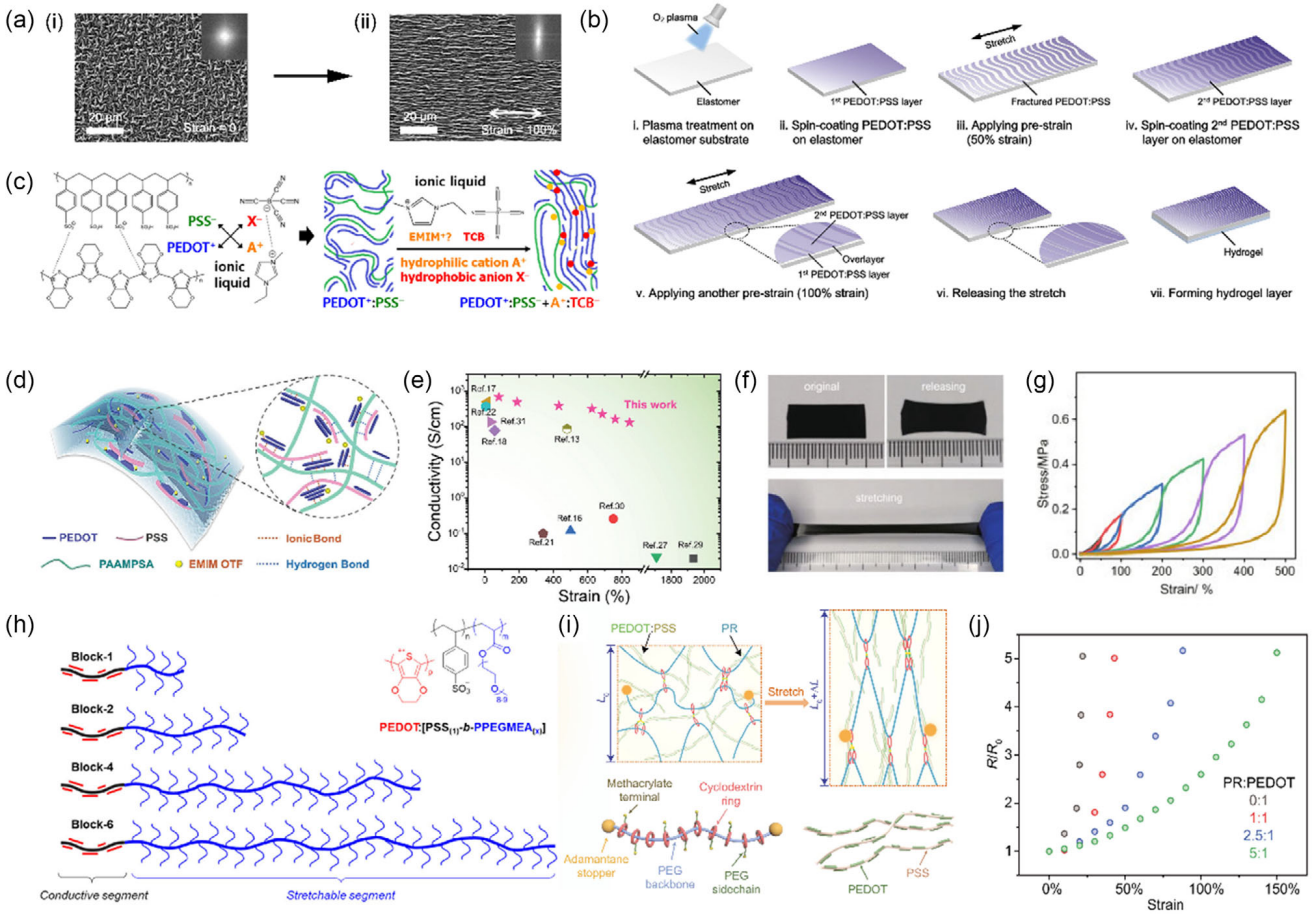
Improve the stretchability of PEDOT:PSS‐based electrodes. a) Morphology of the electrode under 0% (i) and 100% (ii) strain observed by scanning electron microscopy (SEM). The insets are the Fourier transform images. Reproduced with permission.^[^ ^91^ ^]^ Copyright 2019, American Chemical Society. b) Schematic illustration of the fabrication process of the PEDOT:PSS strain sensor. Reproduced with permission.^[^ ^92^ ^]^ Copyright 2021, Elsevier. c) Ion‐exchange mechanism of PEDOT:PSS induced by IL. Reproduced with permission.^[^ ^103^ ^]^ Copyright 2022, American Chemical Society. d) Schematic demonstration of PEDOT:PSS/PAAMPSA/IL composites. e) Comparison of PEDOT:PSS/PAAMPSA/IL with reported representative stretchable conductors. d,e) Reproduced with permission.^[^ ^108^ ^]^ Copyright 2022, Wiley‐VCH. f) Photographs of the polymers in original state and under 400% strain. g) Stress–strain curves of the PEDOT:PSS films under cyclic tests. Reproduced with permission.^[^ ^109^ ^]^ Copyright 2022, Springer Nature. h) Increasing PEDOT:[PSS_(1)_‐*b*‐PPEGMEA_(*x*)_] block copolymers with PPEGMEA (blue) attached to PSS (black). Reproduced with permission.^[^ ^110^ ^]^ Copyright 2022, American Chemical Society. i) Schematic illustration of the PR network. *L* is the length of the film. j) Resistive change–strain curves of different TopoE films. i,j) Reproduced with permission.^[^ ^111^ ^]^ Copyright 2022, American Association for the Advancement of Science.

Similar to the inorganic materials‐based stretchable electronics, the snake, “island bridge” and buckling structure can improve the stretchability of devices. Vosgueritchian et al. deposited PEDOT:PSS doped with Zonyl FS‐300 on PDMS with 15% prestrain. The PEDOT:PSS/PDMS sample was able to undergo 5000 stretching cycles under 10% strain.^[^ ^90^ ^]^ Li et al. transferred PEDOT:PSS onto PDMS with biaxial prestrain, enabling the electrode to withstand strain up to 100%.^[^ ^91^ ^]^ As shown in **Figure** **5**a, the great extensibility is attributed to the introduced folds and wrinkles on surface. Inspired by the scaly skin of reptiles, Liu et al. first deposited a layer of PEDOT:PSS on elastomer substrate, and then deposited another layer of PEDOT:PSS after applying 50% prestrain to the substrate to form a scale‐like imbricate structure, which improved the stretchability of the electrodes (Figure 5b).^[^ ^92^ ^]^ It was successfully applied as a strain sensor with a wide detection range. Bandodkar et al. printed serpentine PEDOT:PSS electrodes on Ecoflex, which could withstand 100% strain with little loss of electrochemical performance.^[^ ^93^ ^]^ Among the approaches based on geometric configuration design, depositing/transferring PEDOT:PSS films onto elastomers needs to pay particular attention to the contact stability between films and elastomers, which will be discussed in the next section.

#### 3.2.2 Blending with Plasticizers, Ionic Liquid, and Polymers

The addition of plasticizers can weaken the interaction between polymer chains and increase the free volume, thereby reducing the elastic modulus of material and improving the extensibility of material. Plasticizers commonly used in PEDOT:PSS are xylitol, glycerol, Triton X‐100, and ILs.^[^ ^94^, ^95^, ^96^, ^97^ ^]^ Savagatrup et al. studied the effect of DMSO and Zonyl FS‐300 on the conductivity and mechanical behavior of PEDOT:PSS. When 10 wt% Zonyl FS‐300 and 5 wt% DMSO were added, PEDOT:PSS films could withstand 28% strain due to plasticization effect.^[^ ^98^ ^]^ Ouyang group found that adding d‐sorbitol can improve both the stretchability and conductivity of PEDOT:PSS. The fracture strain of PEDOT:PSS could be increased to more than 60%, and Young's modulus and tensile strength would decrease with the increase of d‐sorbitol.^[^ ^99^ ^]^ The plasticizer mechanism lies in the interaction with PSSH chains. The interactions between PSSH chains are mainly hydrogen bonds. The hydroxyl group of D‐sorbitol can form hydrogen bonds with PSSH, resulting in the destruction of the hydrogen bonds between PSSH chains. Also, d‐sorbitol can act as lubricant to promote the dissociation and sliding of PEDOT:PSS under strain.

Doping PEDOT:PSS with ILs is a common method to obtain high conductivity and fracture strain simultaneously. Bao group induced morphological changes of PEDOT:PSS by doping IL with strong acidic anions, which can soften PSS and promote better bonding and higher crystallinity between PEDOT.^[^ ^100^ ^]^ The conductivity can exceed 4100 S cm^−1^ at 100% strain. Hydration of IL‐doped PEDOT:PSS can transform it into hydrogel with low Young's modulus, which has been successfully used for electrical stimulation of mouse sciatic nerves.^[^ ^101^ ^]^ Li et al. found that the addition of bis(trifluoromethane)sulfonimide lithium salt (LiTFSI) could simultaneously enhance the thermoelectric and mechanical properties of PEDOT:PSS.^[^ ^102^ ^]^ Based on the theory of hard and soft acids and bases, Choi et al. proposed that the ILs, composed of hydrophilic hard cations and hydrophobic soft anions, could induce phase separation between PEDOT and PSS more effectively (Figure 5c). It was proved by DFT calculation and molecular dynamics simulation.^[^ ^103^ ^]^

Blending conductive materials with polymers/elastomers is a common strategy to improve stretchability. Due to its water solubility, blending PEDOT:PSS with water‐soluble polymers such as PVA, polyethylene oxide (PEO), and PEG has been extensively studied.^[^ ^104^, ^105^ ^]^ Ouyang group systematically studied the influence of polymer blending with different relative molecular masses on mechanical properties. They found that blending PEDOT:PSS with lower crystallinity polymer was conducive to improving the fracture strain. In addition, the researchers blended PEDOT:PSS with waterborne polyurethane (WPU) to increase the toughness of PEDOT:PSS. Blending with water‐insoluble polymers such as PDMS is more difficult.^[^ ^106^ ^]^ Noh considerably improved the compatibility between PEDOT:PSS and PDMS by adding amphiphilic block copolymer PDMS‐*b*‐PEO.^[^ ^107^ ^]^ With the increase of PDMS‐*b*‐PEO, the two phases gradually mixed and finally formed the network structure. Recently, Su et al. blended the soft polymer [poly(2‐acrylamido‐2‐methyl‐1‐propanesulfonic acid (PAAMPSA)] with PEDOT:PSS, whose stretchability could be as high as 630%. At the same time, they enhanced its conductivity by adding 1‐ethyl‐3‐methylimidazolium trifluoromethanesulfonate (EMIM OTF).^[^ ^108^ ^]^ As shown in Figure 5d, the formation of hydrogen bonds and ionic bonds could dissipate energy by bond breaking when subjected to strain or damage. In addition to, the dynamic bonds enabled the self‐healing properties. The effect of different proportions of PAAMPSA on conductivity and stretchability of the complex was also investigated. With the increase of PAAMPSA, the stretchability increased whereas conductivity decreased due to the decrease of PEDOT. Therefore, the appropriate amount of PAAMPSA should be optimized according to specific requirements (Figure 5e). Tan et al. obtained conductive polymers with low modulus (56.1–401.9 kPa), high stretchability (700%), and high interface adhesion (>1.2 MPa) by using supramolecular solvents (β‐cyclodextrin and citric acid) as dopants and PVA as cross‐linking network.^[^ ^109^ ^]^ As shown in Figure 5f,g, the addition of PVA network reduces the residual strain of the polymer. When the mass ratio of PEDOT:PSS was 3.6 wt%, the reversible elongation strain was 400%. Under larger tensile strain, the residual strain was less than 50%. Blau et al. introduced poly(poly(ethylene glycol) methyl ether acrylate) (PPEGMEA) to PEDOT:PSS to synthesize PEDOT:PSS‐*b*‐PPEGMEA_(X)_ block polymers (Figure 5h). The effect of PPEGMEA length on the mechanical properties of PEDOT:PSS was investigated in detail. Increasing the length of the PPEGMEA chain not only decreased the elastic modulus and tensile strength of polymer, but also increased the fracture strain and toughness.^[^ ^110^ ^]^ The loss of conductivity can be recovered by blending with PEDOT:PSS. Jiang et al. creatively designed the topological supramolecular network (PR) and introduced it into PEDOT:PSS to form a “mechanical interlocking structure” with high conformational degrees of freedom. As shown in Figure 5i, the PR network was composed of PEG backbone and sliding cyclodextrins (CDs) functionalized with poly(ethylene glycol) methacrylate (PEGMA) side chains, conferring this polymer a high intrinsic stretchability up to 150% (Figure 5j).^[^ ^111^ ^]^ In addition to, the polar PEG chain segment of PR can partially replace PSS and enhance the aggregation of PEDOT, thus further improving the conductivity.

### 3.3 Improving Stability of Poly(3,4‐Ethylenedioxythiophene):Poly(Styrenesulfonate) Electrodes

**Figure 6:**
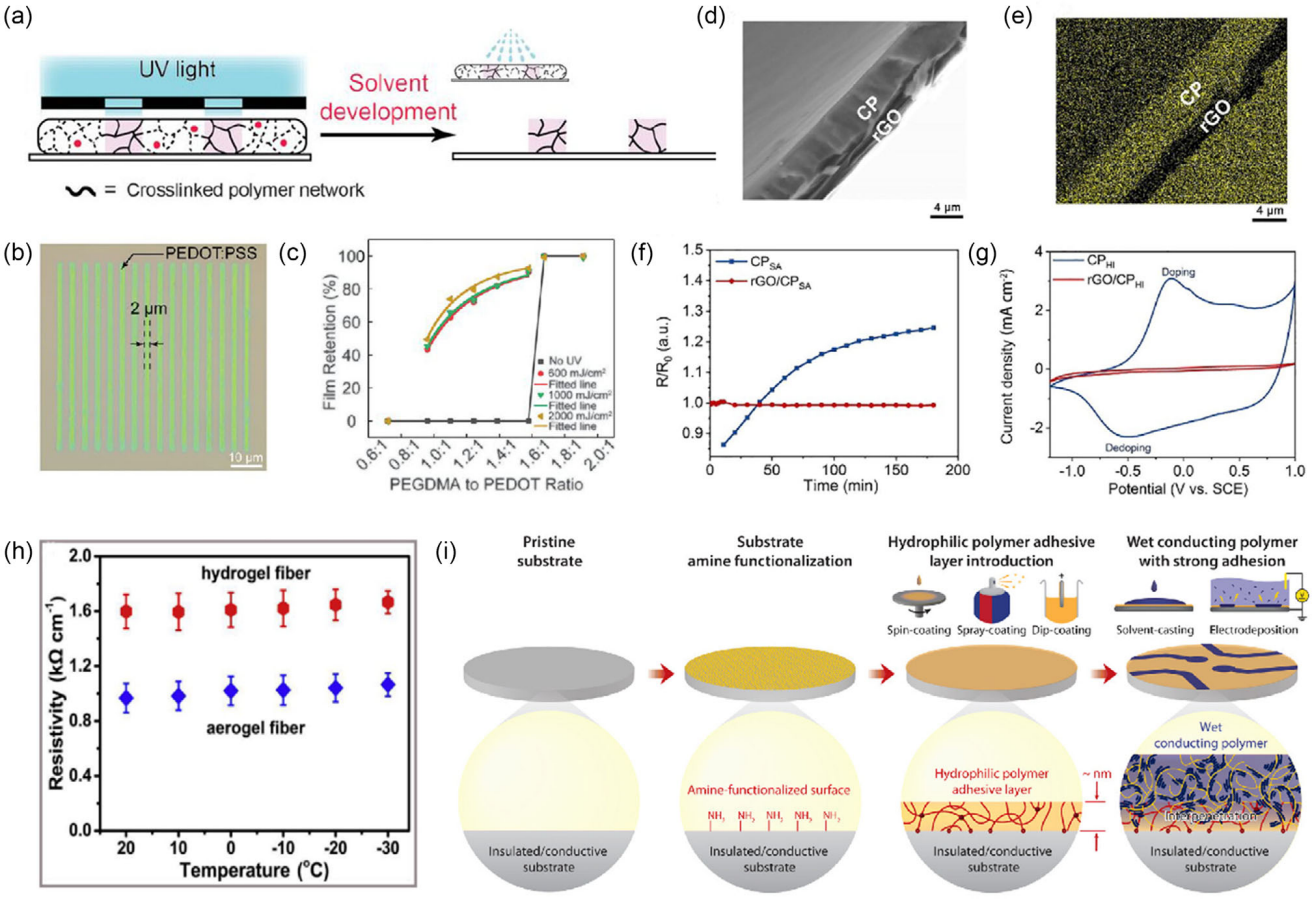
Improving the stability of PEDOT:PSS‐based electrodes. a) Schematic illustration of the mechanism using direct optical lithography strategy.^[^ ^127^ ^]^ b) Optical image of PEDOT:PSS line pattern with a resolution of 2 μm.^[^ ^127^ ^]^ c) Gel curves of different PEDOT:PSS under different UV doses. a–c) Reproduced with permission.^[^ ^127^ ^]^ Copyright 2021, American Association for the Advancement of Science. d,e) Cross‐sectional SEM (d) and EDS (e) images of the rGO/PEDOT:PSS‐based film. f) Resistance changes–time curves of the PEDOT:PSS only (CP) and rGO/CP films in water. g) CV curves of CP and rGO/CPHI electrodes in a buffer saline. d–g) Reproduced with permission.^[^ ^129^ ^]^ Copyright 2021, Elsevier. h) Plots of resistivity of hydrogel fiber and aerogel fiber in the range of −30 to 20 °C. Reproduced with permission.^[^ ^129^ ^]^ Copyright 2021, Elsevier. i) Preparation process of wet conducting polymer on amine‐functionalized substrate. Reproduced with permission.^[^ ^131^ ^]^ Copyright 2020, American Association for the Advancement of Science.

The stability of electrode is one of the vital factors for bioelectrical signal monitoring. For PEDOT:PSS, the main consideration is its stability in humid environment and adhesion stability with elastic substrate. Due to the hygroscopicity of PSS, PEDOT:PSS film will absorb water from surrounding environment and swell, which is detrimental to the structural stability of PEDOT:PSS. Many researches have been devoted to solving this problem. For example, researchers reduced the water absorption of PEDOT:PSS films by posttreatment with solvents such as EG and acid.^[^ ^112^, ^113^ ^]^ The principle is removing a large amount of PSS/PSSH, thereby increasing their stability in humid environment. Some researchers have also enhanced the water resistance of PEDOT:PSS by adding additives, such as IL, difluorobenzene azide, *N*‐methylol acrylamide, butyl acrylate, and carboxymethylcellulose sodium.^[^ ^114^, ^115^, ^116^, ^117^ ^]^ Jorge et al. added 3‐methyl‐3‐oxetanemethanol to PEDOT:PSS to enhance its conductivity and underwater stability simultaneously.^[^ ^118^ ^]^ Physical cross‐linking method can also improve the stability of PEDOT:PSS in water.^[^ ^119^, ^120^ ^]^ For instance, the water resistance of PEDOT:PSS could be improved by forming a cross‐linking network with additives through photopolymerization or thermal polymerization. The (3‐glycidyloxypropyl)trimethoxysilane (GOPS) is a widely used chemical cross‐linker for PEDOT:PSS.^[^ ^121^, ^122^, ^123^ ^]^ Hakansson et al. found that by adding 0.1 v v^−1^% GOPS in PEDOT:PSS solution, the PEDOT:PSS would maintain good adhesion to the glass substrate. This was due to the generation of a triple cross‐linking network: GOPS‐GOPS, GOPS‐PSS, and GOPS‐glass. However, the conductivity of PEDOT:PSS was decreased.^[^ ^124^ ^]^ ElMahmoudy et al. investigated an optimum concentration of GOPS in PEDOT:PSS to maintain the electrical performance while improving the film stability.^[^ ^125^ ^]^ In addition to GOPS, Ha et al. introduced 2‐pentacosa‐10,12‐diynamidoethylsulfate (PCDSA) monomer.^[^ ^119^ ^]^ Agua et al. introduced divinyl sulfone (DVS).^[^ ^126^ ^]^ Recently, Bao group introduced poly(ethylene glycol) dimethacrylate (PEGDMA) and topological polymer network to form a double network with PEDOT:PSS,^[^ ^111^, ^127^ ^]^ making it compatible with traditional optical lithography technique (**Figure** **6**a–c). Water was used as the developer and PEDOT:PSS electrode arrays with a resolution as low as 2 μm can be obtained. The reaction mechanism is shown in Figure 6a. In addition, by virtue of the compactness of graphene/reduced graphene oxide (rGO) films, Yao et al. coated PEDOT:PSS with a layer of GO, which was then reduced to rGO (Figure 6d,e)^[^ ^128^ ^]^ to protect PEDOT:PSS from being dedoped by alkali and biological reducing agents through molecular repulsion. rGO films can not only enhance the environmental stability of PEDOT:PSS, but also improve the overall electrical conductivity through the bridging effect from its large conjugate domain. Figure 6f,g shows the real‐time resistance changes of the electrode immersed in water and the cyclic voltammetry (CV) curves in phosphate‐buffered saline (PBS) solution, respectively. The rGO/PEDOT:PSS electrodes showed great stability in both water and PBS solution. Recently, some studies focused on the stability of PEDOT:PSS at low temperatures. Xu et al. prepared graphene/PEDOT:PSS hydrogel fibers and then introduced the antifreezing PVA network into the hydrogel fibers by solvent replacement. Finally, antifreezing stretchable graphene/PEDOT‐PVA hydrogel fibers with double networks were prepared (Figure 6h).^[^ ^129^ ^]^

PEDOT:PSS films are usually placed on elastic substrates to be epidermal/implanted sensing electrodes. When human skin/tissue undergoes strain, PEDOT:PSS is prone to detach from substrate, resulting in signal shift or complete loss. Therefore, various strategies have been used to improve the adhesion stability between PEDOT:PSS and substrate, such as adding a bonding layer between them and giving special treatment to substrate. Liu et al. increased the adhesion of electrodes by introducing polyethylenimine ethoxylated (PEIE) onto electrode surface. The mechanism lies in the covalent cross‐linking between PEIE and PSS.^[^ ^130^ ^]^ Zhao group developed a bonding layer that allowed conductive polymer gels to be attached to a variety of commonly used substrate materials.^[^ ^131^ ^]^ As shown in Figure 6i, amino groups were modified on the substrate and then the bonding layer (hydrophilic polyurethane) was coated onto the substrate. Since the bonding layer was also hydrophilic, the conductive polymer precursor would diffuse into the bonding layer after swelling, forming an interpenetrating network. Therefore, it would not dissociate in humid environment. Zheng et al. treated poly(ethylene terephthalate) (PET) substrate with oxygen plasma and ethanol, through which more hydrogen bonds were formed between PSS and substrate to increase adhesion.^[^ ^132^ ^]^ Ganji et al. improved adhesion by increasing the roughness of substrate. Compared with gold film substrate, gold nanorods substrate showed higher adhesion.^[^ ^133^ ^]^ In addition, amine‐functionalized EDOT‐NH_2_ can also be synthesized on substrate surface by electrical grafting, and then EDOT‐NH_2_ copolymerized with EDOT to form conjugated polymer, which was covalently bonded to the substrate, thereby enhancing the adhesion of PEDOT film and allowing it to be used as an implanted electrode.^[^ ^134^ ^]^

## 4 Poly(3,4‐Ethylenedioxythiophene):Poly(Styrenesulfonate) Bioelectrodes for Sensing Bioelectrical Signals

Bioelectrical signals are basic physiological signals of human beings. Common bioelectrical signals include EMG (50 μV–50 mV), ECG (1–5 mV), EOG (0.01–0.1 mV), and EEG (0.001–0.1 mV),^[^ ^135^ ^]^ which feature low amplitude and low frequency. These characteristics make it susceptible to noise and motion artifacts during collecting. So, the bioelectrode becomes the key to ensure the quality of signals. The main challenge in monitoring bioelectrical signals is the modulation of ion concentrations delivered across the bioelectronic interface. Different from the inorganic material electrodes with only electron–hole transmission, PEDOT:PSS electrodes use ions and electrons as charge carriers to conduct bioelectrical signals directly. According to different working environments, PEDOT:PSS‐based electrodes could be classified as epidermal electrode and implanted electrode. The most recent work of bioelectrical signal sensing with epidermal and implantable PEDOT:PSS‐based electrode will be discussed in the following.

### 4.1 Epidermal Sensing of Bioelectrical Signals

**Figure 7:**
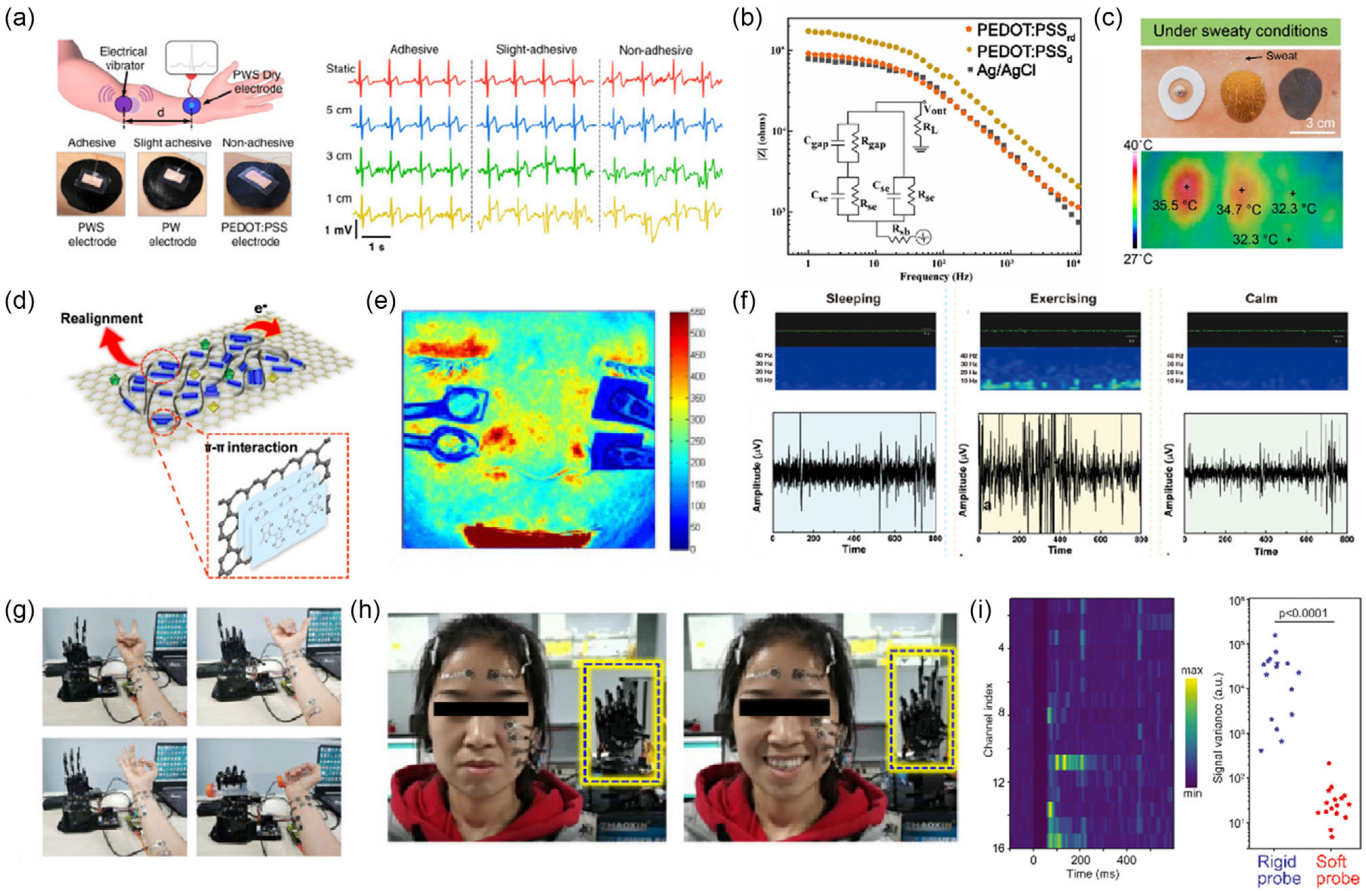
Bioelectrical signals sensing by epidermal PEDOT:PSS bioelectrodes. a) Dynamic ECG recording induced by an electrical vibrator with different distances to the electrode. Reproduced with permission.^[^ ^137^ ^]^ Copyright 2020, Springer Nature. b) Contact impedance curves of three types of electrodes. Reproduced with permission.^[^ ^138^ ^]^ Copyright 2022, Elsevier. c) Optical and infrared photos of various electrodes on skin after exercise. Reproduced with permission.^[^ ^141^ ^]^ Copyright 2021, American Chemical Society. d) Schematic demonstration of synergistic interaction between graphene and PEDOT:PSS. e) The laser speckle contrast imaging of the blood perfusion under PTG electrodes. f) Long‐time monitoring of EEG. Original EEG signals (top) and fast Fourier transform EEG signals (bottom). g) Photograph of five PTG electrode pairs driving the robotic hand to show various gestures. h) Photograph of two PTG electrode pairs driving the robotic hand based on multichannel facial sEMG. d–h) Reproduced with permission.^[^ ^143^ ^]^ Copyright 2021, Springer Nature. The photos in (h) appear in this review with the consent of the subject. i) Left: peristimulus time histogram of EMG activities of the octopus. Right: signal variance recorded by rigid probe and TopoE‐S probe during the resting state. Reproduced with permission.^[^ ^111^ ^]^ Copyright 2022, American Association for the Advancement of Science.

Skin can be regarded as a signal source: it can both generate and transmit biological signals, thereby providing an important index of individual health.^[^ ^136^ ^]^ Conventional epidermal electrode is Ag/AgCl electrode, which maintains good contact with skin through conductive gel and has the advantages of low contact impedance, low cost, and ease to use. However, the gel may irritate the patient's skin. After long‐term use, the gel would undergo dehydration and coagulation, resulting in signal detection noise and low signal quality. To avoid these problems, researchers are devoted to proposing dry electrodes. Ouyang group mixed PEDOT:PSS, WPU, and D‐sorbitol to prepare dry electrodes (PWS) with good self‐adhesion, stretchability, and conductivity.^[^ ^137^ ^]^ The electrodes can obtain ECG, EMG, and EEG signals on both dry and wet skin surfaces. As shown in **Figure** **7**a, an electromechanical vibrator was immobilized on the skin to generate skin shaking. The PWS were able to collect ECG signals at different positions from the vibrator due to their good adhesion. Chen et al. reported a PEDOT:PSS electronic tattoo without substrate. PEDOT:PSS/PVA dry electrodes can transform to gel through gelation on the skin to achieve interface energy regulation and good adhesion.^[^ ^138^ ^]^ As shown in Figure 7b, the skin interface impedance and ECG signal quality of the electronic tattoo were equivalent to those of the Ag/AgCl electrode, yet more comfortable and reliable. Lo et al. blended EG‐doped PEODT:PSS with PEO and prepared polymer films by inkjet printing. The blended polymer showed a low sheet resistance of 84 Ω sq^−1^ and could resist 50% tensile strain.^[^ ^139^ ^]^ It was printed as interconnects of the dry electrode on PDMS to realize the detection of photoplethysmography (PPG) and ECG. Lan et al. embedded a new double network in PEDOT:PSS: physically cross‐linked poly(vinyl alcohol) (PVA) and covalently cross‐linked polyethylene glycol diacrylate (PEGDA) to achieve the synergistic regulation of interpenetration and molecular crystallinity in blends.^[^ ^140^ ^]^ The interaction between PVA and PEGDA made the film maintain well‐defined phase separation. The electrode–skin interface impedance was lower than that of Ag/AgCl electrode, and the quality of ECG signal recorded by this electrode was equivalent to Ag/AgCl electrode. Li et al. embedded PEDOT:PSS into glycerol‐plasticized silk fiber mats and obtained a silk‐based electrode. This electrode can tightly adhere to the skin under high temperature and high humidity conditions while still possesses physiological comfortability (Figure 7c).^[^ ^141^ ^]^ The silk‐based electrode exhibited high stretchability (>250%), low thermal insulation (≈0.13 °C m^2^ W^−1^), low evaporative resistance (≈23 Pa m^2^ W^−1^), and high water vapor transmission rate (≈117 g m^−2^ h^−1^). Adding glycerol and annealing enhanced the stability of the electrode to water and sweat. The high stretchability of the electrode enabled it to record signals stably under a large degree of deformation. The SNR and amplitude of the EMG signals recorded at different deformation lengths were equivalent to those of Ag/AgCl electrode. Yao et al. introduced rGO layer on PEDOT:PSS to improve its stability and used it for EMG signal monitoring and electrical stimulation.^[^ ^128^ ^]^ Similarly, Sinha et al. coated PEDOT:PSS on nonwoven fabrics that were previously coated with graphene/graphite to obtain an electrode with ultra‐low sheet resistance (1.1 Ω sq^−1^) for ECG signal detection.^[^ ^142^ ^]^

In addition to the above bulk electrodes, ultra‐thin electrodes with ultraconformability can resist motion artifacts, thus being applied in various scenes. Using the synergistic interaction between graphene and PEODT:PSS, our group prepared ultra‐thin electrodes (≈100 nm) with excellent conformability to the skin, exhibiting low sheet resistance (≈24 Ω sq^−1^, 4142 S cm^−1^), high transparency, and electrical stability.^[^ ^143^ ^]^ As shown in Figure 7d, this synergistic effect was due to the strong π–π interaction between PEDOT:PSS and graphene, leading to delocalization of π electrons. This induced a high degree of molecules ordering of PEDOT and charge transfer between graphene, thus improving the charge carrier mobility of PEDOT:PSS transferred CVD graphene (PTG) films as a whole. The PTG electrode is successfully used for facial muscle assessment, with less interference during testing compared to the Ag/AgCl electrode. For extremely weak EEG signals, it also showed stability during long‐term monitoring (Figure 7e,f). In addition, PTG electrodes have been successfully applied in HMI. As a way of communication between people and external devices, HMI transforms virtual ideas into actions of machine, which is widely used in robot control, flight control, VR/AR, and other fields.^[^ ^144^ ^]^ As shown in Figure 7g, five pairs of PTG electrodes were adhered to the designated positions to obtain the sEMG signals of thumb, index finger, middle finger, ring finger, and little finger and remotely operated the movements of robot hands. As shown in Figure 7h, two pairs of PTG electrodes were attached to specific locations on the face to control index and middle fingers. The robot hands can make different gestures according to the facial expressions of volunteers. Jiang et al. prepared high‐density stretchable electrode arrays by introducing topological supramolecular networks into PEDOT:PSS to record EMG signals with high spatial and temporal resolution.^[^ ^111^ ^]^ Because of its excellent stretchability, the electrode can be placed on octopus to detect EMG signals. As shown in Figure 7i, the signal collected by this electrode had a higher SNR than that collected by PEDOT:PSS/polyimide (PI) electrode. Won et al. prepared PLA/PEDOT:PSS laminated electrodes and formed the Y‐shaped kirigami patterns with an area coverage of 85% by laser cutting. They applied the electrodes to detect EMG, ECG, and EOG signals and successfully used EOG to control the switches of household appliances.^[^ ^145^ ^]^

PEDOT:PSS films can serve as the active layer channel in the organic electrochemical transistors (OECTs), which feature high transconductance to amplify signals.^[^ ^146^, ^147^, ^148^ ^]^ In 2017, Braendlein et al. first demonstrated the voltage‐to‐voltage amplifying transduction of ECG signals with an OECT. They combined the OECT‐based amplifier with the medical electrodes, and built a circuit to produce voltage output. It showed that the OECT provided increased sensitivity in the saturation regime while maintaining a linear signal transduction.^[^ ^149^ ^]^ This OECT had no direct contact with skin. The fundamental operation of OECTs requires an electrolyte‐rich environment, while skin does not offer enough electrolyte. Recently, Spyropoulos et al. innovatively developed an internal ion‐gated OECT (IGT), which provided a comfortable interface with skin. By using a hybrid PEDOT:PSS/d‐sorbitol layer as the channel, the supply of ions within the conducting polymer was maintained, which enabled the IGTs to work efficiently on skin to record EEG signals without external ionic sources.^[^ ^150^ ^]^

### 4.2 In Vivo Sensing of Bioelectrical Signals

**Figure 8:**
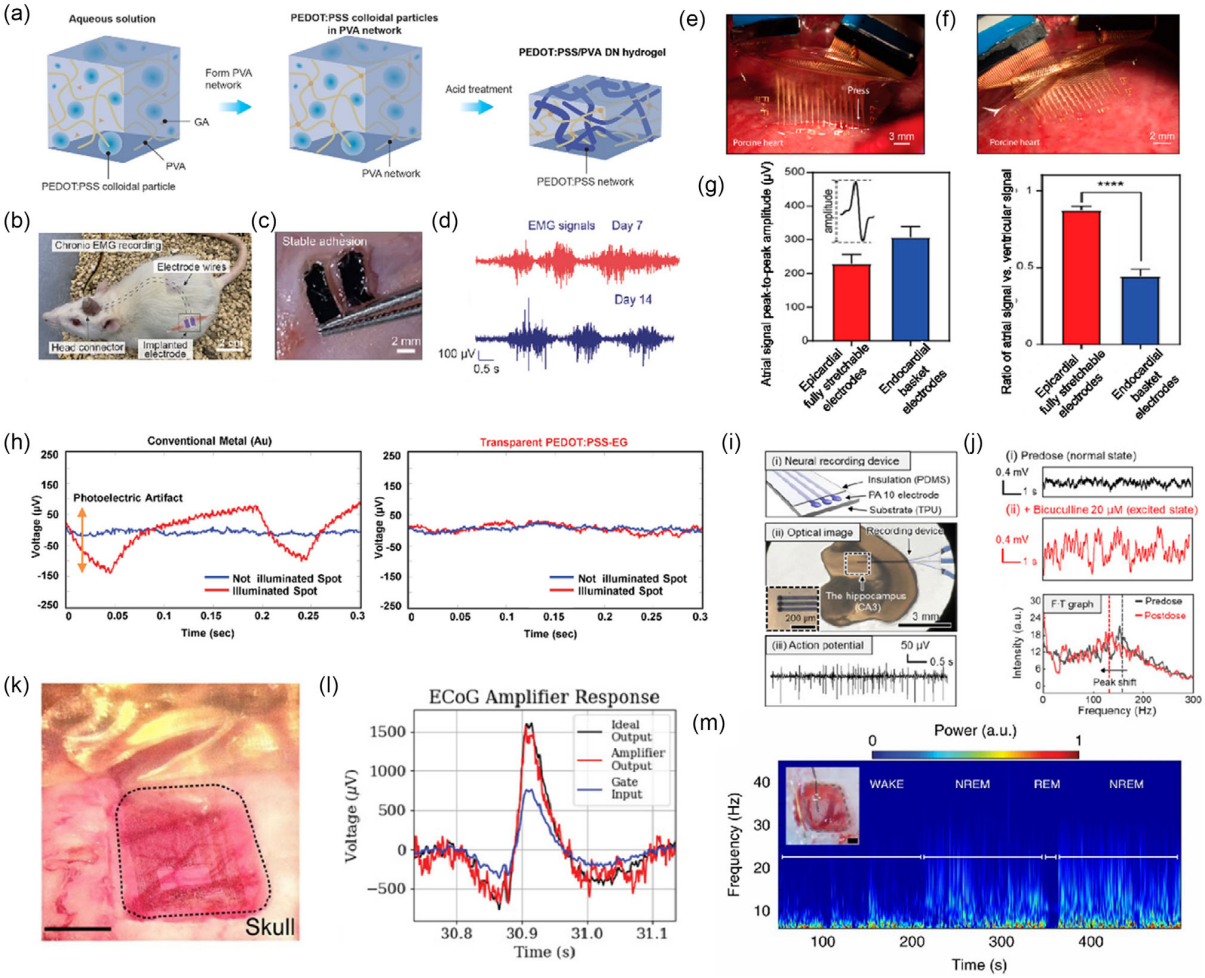
Bioelectrical signals by implantable PEDOT:PSS bioelectrodes. a) Schematic demonstration of the fabrication process of PEDOT:PSS and PVA composites. b) Photograph of a rat when recording chronic EMG. c) Photograph of a pair of conducting hydrogel electrodes stably adhered on muscle. d) EMG signals recorded by the hydrogel electrodes after 7 and 14 days of implantation. a–d) Reproduced with permission.^[^ ^151^ ^]^ Copyright 2022, Wiley‐VCH. e,f) Optical image of the array in partial contact e) and full contact f) with the porcine right ventricle (RV). g) Statistical analysis of the amplitude (left) and the ratio (right) of the atrial signal to the ventricular signal from both epicardial and endocardial electrodes. e–g) Reproduced with permission.^[^ ^153^ ^]^ Copyright 2020, National Academy of Sciences, USA. h) Photoelectric artifacts of illuminated (red) and not illuminated (blue) spots. Reproduced with permission.^[^ ^154^ ^]^ Copyright 2021, Wiley‐VCH. i) Schematic illustration shows the recording device on mouse brain slice and extracellular AP from hippocampus (CA3). j) LFP signal data recorded during normal state and excited state. i,j) Reproduced with permission.^[^ ^88^ ^]^ Copyright 2022, American Association for the Advancement of Science. k) Optical image of the ECoG probe on the somatosensory cortex. The craniotomy is surrounded by dashed lines. Scale bar: 1 mm. Reproduced with permission.^[^ ^155^ ^]^ Copyright 2013, Springer Nature. l) ECoG amplifier response data. Reproduced with permission.^[^ ^156^ ^]^ Copyright 2021, Wiley‐VCH. m) Time–frequency spectrogram of chronic LFP recording with behavioral states marked. Inset shows a photograph of e‐IGT‐based device placement on rat cortex; scale bar, 500 μm. Reproduced with permission.^[^ ^157^ ^]^ Copyright 2020, American Association for the Advancement of Science.

Monitoring electrical signals in vivo is more challenging. The organ/tissue surface is more irregular, the elastic modulus is lower (<10 kPa), and the internal environment is more complex. Thus, the demand for electrode compatibility, stability, and signal output quality is higher. Conventional implanted electrodes are made of noble metals and silicon, which have good electrical conductivity but much higher Young's modulus (≈100 GPa) than human tissue.^[^ ^42^ ^]^ This mechanical mismatch would not only lead to the poor detection signal quality, but also tissue damage and neuroinflammation. Compared with conventional electrode, PEDOT:PSS‐based hydrogel electrodes have lower Young's modulus, which could be mechanically match with organ/tissue. Guo group reported a PEDOT:PSS/PVA‐based double‐network hydrogel with high conductivity (≈10 S cm^−1^), large fracture strain (≈150%), and low Young's modulus (≈460 kPa).^[^ ^151^ ^]^ As shown in **Figure** **8**a, the relative mass ratio of PEDOT:PSS to PVA in the precursor hydrogel was significantly increased by reducing the content of PVA. Then, the acid treatment was used to construct a highly conductive polymer network. The solid components were concentrated by dehydration to form a compact network structure. Two PEDOT:PSS/PVA conductive hydrogel electrodes were completely implanted into the right biceps femoris muscle of a rat for chronic EMG recordings (Figure 8b,c). According to the EMG of rats on days 7 and 14, the EMG signals were similar in signal intensity and noise level, indicating that the electrode performance and tissue–electrode interface were stable during long‐term implantation (Figure 8d). In addition, the hydrogel electrode was successfully applied to rat sciatic nerve electrical stimulation. Wei et al. prepared an intelligent cloaking gold‐coated nanofiber mesh (Au_c_‐NM)/PEDOT:PSS device. The hydrogel contact lens device had excellent optical transparency, mechanical compliance, stability, and high gas permeability.^[^ ^152^ ^]^ Researchers placed it on rabbit eyes to record electroretinogram (ERG). The electrode successfully recorded ERG signals under various conditions, including scotopic potential responses at different light intensities, scotopic oscillatory potentials, photopic potential responses, and photopic 30 Hz flicker ERG responses.

Multielectrode array features high resolution and high integration, which can obtain more information than single‐channel electrode. Liu et al. prepared a PEDOT:PSS microelectrode array with cell‐level resolution by using optical lithography technology and placed it on a porcine heart model with chronic atrial fibrillation for epicardial mapping.^[^ ^153^ ^]^ The electrode can be well attached to the dynamically beating heart and stably recorded ECG signals (Figure 8e,f). This stretchable microelectrode array showed twofold higher atrium–ventricular signal ratio and greater than 100‐fold spatial resolution compared with the most advanced endocardial mapping technique (Figure 8g). PEDOT:PSS can be used in optogenetics due to its excellent transparency. Cho et al. prepared the PEDOT:PSS electrode array by optical lithography technology. The conductivity of this electrode was improved after EG treatment, and the impedance was lower than that of the bilayer graphene electrode.^[^ ^154^ ^]^ When used for ECoG recordings, it showed smaller optical artifacts and higher sensitivity compared with the semitransparent gold film electrode. As shown in Figure 8h, under the same power laser stimulation, ≈200 μV electrical signal noise was recorded by Au electrode, while only ECoG signals could be recorded by the transparent PEDOT:PSS electrode. Won et al. used a novel process to prepare PEDOT:PSS hydrogels by laser patterning, which would induce phase separation between PEDOT and PSS.^[^ ^88^ ^]^ To enhance the interaction between laser and PEDOT:PSS, AuNPs were added. They obtained an underwater stable PEDOT:PSS hydrogel with high conductivity (670 S cm^−1^) and high resolution (6 μm). As shown in Figure 8i, this electrode was first used to record extracellular APs of mouse brain slices, and stable continuous spikes were obtained. Then, the electrode was used to record in situ local field potential (LFP). The changes in LFP signal amplitude before and after brain excitation could be clearly recorded attributable to the good conductivity and spatial resolution of the electrode (Figure 8j). The electrode can also be used for electrical stimulation of the sciatic nerve in mice due to its great charge storage capacity (CSC).

In recent years, OECT has become a focus in the area of in vivo bioelectronics. The high transconductance endows OECTs a large amplification capability and a low limit of detection, and they can operate in aqueous environment with high stability.^[^ ^146^, ^148^ ^]^ Khodagholy et al. first demonstrated OECT arrays as ECoG probes. As shown in Figure 8k, the probes were placed on somatosensory cortex of a rat. The results showed that the OECT exhibited a much better SNR (44 dB) than iridium penetrating electrodes and surface electrodes (24.3 dB).^[^ ^155^ ^]^ Tyrrell et al. considered viable combinations of voltage and resistor values and successfully implemented the liner amplifier circuit with an OECT as the amplifying element. Three prerecorded electrophysiological signals were input and with the amplification of frequency filters, ECoG and SD inputs showed greater efficacy in amplification (Figure 8l).^[^ ^156^ ^]^ Cea et al. developed an enhancement‐mode, internal ion‐gated OECT (e‐IGT), which enabled long‐term stable operation. The e‐IGT channel was composed of PEDOT:PSS, polyethylenimine (PEI), d‐sorbitol, and crosslinking additives. e‐IGTs arrays were placed on the cortical surface and performed chronic brain recording. After implantation for 2 weeks, the local field potentials were recorded. The corresponding time–frequency spectrogram is shown in Figure 8m, from which the nonrapid eye movement (NREM) sleep and rapid eye movement (REM) were identified. This e‐IGTs showed excellent stability in biological environments.^[^ ^157^ ^]^

## 5 Summary and Perspectives

PEDOT:PSS, as one of the most representative conductive polymers, has received extensive attention since commercialization. Its mixed ionic and metallic conductivity, transparency, ductility, biocompatibility, and ease of processing make it an ideal candidate for bioelectronic interfaces. Monitoring bioelectrical signals is a significant approach to understand human health status. To acquire high quality bioelectrical signals continuously, PEDOT:PSS‐based bioelectrodes need to be further modified. In this review, we first introduce the generation mechanism and the characteristics of bioelectrical signals. The difficulties in detection are illustrated. Thereafter, the preparation requirements of bioelectrodes and the strategies to enable PEDOT:PSS as bioelectrodes are discussed. Finally, we overview the developments of PEDOT:PSS‐based electrodes in bioelectrical signal sensing (**Table** **1**) by listing representative works in recent years.

**Table 1:** Summary of PEDOT:PSS bioelectrodes for sensing bioelectrical signals

Materials	Conductivity/Resistivity	Stretchability [%]	Impedance	Modulus	References
PEDOT:PSS/IL/graphene	4142 S cm^−1^/24 Ω sq^−1^	≈30	≈32 kΩ at 100 Hz	640 kPa	[137]
PEDOT:PSS/HI/rGO	≈630 S cm^−1^/1.32 Ω sq^−1^	–	≈2 kΩ cm^2^ at 100 Hz	0.7–1.4 GPa	[128]
PEDOT:PSS/WPU/d‐sorbitol	545 S cm^−1^	43	≈82 kΩ cm^2^ at 10 Hz	≈16 MPa	[138]
PEDOT:PSS/PVA	≈200 S cm^−1^	≈32.5	65.3 kΩ at 10 Hz	15 MPa	[140]
PEDOT:PSS/EG/PEO	84 Ω sq^−1^	50	–	–	[141]
PEDOT:PSS/PEGDMA/PVA	442 S cm^−1^	≈45	≈32 kΩ cm^2^ at 100 Hz	16 MPa	[142]
PEDOT:PSS/silk/glycerol	≈24 S cm^−1^	≈250	≈60 kΩ at 100 Hz	<3 MPa	[143]
PEDOT:PSS/polyrotaxane (PR)/H_2_SO_4_	2700 S cm^−1^ initial ≈6000 S cm^−1^ at 100 strain	150	≈200 Ω at 100 Hz	≈800 MPa	[111]
PEDOT:PSS/PLA	150 Ω sq^−1^	33	≈10 kΩ at 100 Hz	–	[145]
PEDOT:PSS/PVA	10 S cm^−1^	≈150	32.5–35.1 Ω at 1–10 000 Hz	≈460 kPa	[151]
PEDOT:PSS/AuNPs	670 S cm^−1^	20	≈1.1 kΩ at 100 Hz	57 MPa	[88]

In general, electrodes for sensing bioelectrical signals should have the following characteristics: high conductivity, stretchability, adhesion stability, and biocompatibility, and the modification strategies of PEDOT:PSS mainly focus on these three aspects: 1) Conductivity: Improving the conductivity of PEDOT:PSS is mainly achieved by secondary doping or posttreatments. The principle can be interpreted as removing excessive insulate PSS chain segments, inducing phase separation between PEDOT and PSS, and making the conformation of PEDOT becomes more extended or transforms from coil to linear structure. It can improve the effective lamellar stacking of PEDOT and promote charge transport between chains. 2) Stretchability: Strategies to improve the stretchability of PEDOT:PSS include plasticizing or blending with soft polymers/elastomers. The plasticizing mechanism can be attributed to the hydrogen bonds breaking between PSSH chains. In addition, plasticizing can also induce secondary doping of PEDOT:PSS to improve conductivity. 3) Stability: The stability of PEDOT:PSS can be understood as adhesion stability to substrate and water resistance stability. To improve the adhesion stability, adding an adhesive layer between substrate and electrode, designing functional substrates that can achieve dynamic bonding or have special structures are common strategies. By introducing double networks and combining them with well‐sealed graphene can effectively enhance the water stability.

Although many achievements have been made in bioelectrical signal sensing by PEDOT:PSS‐based electrodes, there still remain profound challenges before practical application: 1) Conductivity and biocompatibility: The conductivity of PEDOT:PSS is still inferior to indium tin oxide (ITO), metals, and other materials. At present, the conductivity of PEDOT:PSS is mainly improved by doping and posttreatments. As common solvents used in these methods, ILs are proved to be toxic to human and environment, and strong acids can hurt skin. When applied as bioelectrodes, the potential leakage of ILs and residual of acid can be harmful to human health. Therefore, it is urgent to develop more environmentally friendly and safe strategies, such as increasing the proportion of PEDOT in PEDOT:PSS. 2) Balance between conductivity and mechanical properties: Adding additives in PEDOT:PSS also has the risk of leakage. When blending with intrinsically insulate soft polymers/elastomers, the conductivity of PEDOT:PSS will decrease. Therefore, how to balance conductivity and mechanical properties on the basis of ensuring safety is a problem that needs to be considered. At present, a developing idea is to directly design block copolymers based on PEDOT:PSS. 3) Long‐term stability: PEDOT:PSS is sensitive to humidity and temperature. In addition, the adhesion stability between electrode and skin is necessary for obtaining long‐term and high‐quality bioelectrical signals. To overcome the stability limitations, adequate strategies such as direct cross‐linking, introducing double networks, and self‐doping are in urgent need. When applied in vivo, diversified encapsulation strategies for the electrodes need to be developed to adapt the complex environment. As for OECTs, though there is no need for excessive encapsulation, the high gate voltages required to keep PEDOT:PSS in off‐state are deemed as a threat to its long‐term stability. 4) Electrode size: On one hand, although various bioelectronic interfaces have been developed to communicate with skin/tissue, the size of these systems is usually limited to a few square centimeters, which makes it hard to collect bioelectrical signals over a large area, resulting in certain limitations for whole scalp or back detection. On the other hand, high‐spatial resolution devices need to be further developed for areas that require precise detection, such as brain regions or atria. For example, to monitor the neurotransmitter releasement within the micrometer scale, the dimension of OECT array should be further reduced. Therefore, it is urgent to design fabrication schemes compatible with optical lithography or propose new fabrication schemes to construct multichannel devices.

## Conflict of Interest

The authors declare no conflict of interest.
